# FLT3 mutated acute myeloid leukemia: 2021 treatment algorithm

**DOI:** 10.1038/s41408-021-00495-3

**Published:** 2021-05-27

**Authors:** Naval Daver, Sangeetha Venugopal, Farhad Ravandi

**Affiliations:** Department of Leukemia, The University of Texas – MD Anderson Cancer Center, Houston, TX USA

**Keywords:** Acute myeloid leukaemia, Targeted therapies

## Abstract

Approximately 30% of patients with newly diagnosed acute myeloid leukemia (AML) harbor mutations in the fms-like tyrosine kinase 3 (*FLT3*) gene. While the adverse prognostic impact of *FLT3*-ITD^mut^ in AML has been clearly proven, the prognostic significance of *FLT3*-TKD^mut^ remains speculative. Current guidelines recommend rapid molecular testing for *FLT3*^mut^ at diagnosis and earlier incorporation of targeted agents to achieve deeper remissions and early consideration for allogeneic stem cell transplant (ASCT). Mounting evidence suggests that *FLT3*^mut^ can emerge at any timepoint in the disease spectrum emphasizing the need for repetitive mutational testing not only at diagnosis but also at each relapse. The approval of multi-kinase FLT3 inhibitor (FLT3i) midostaurin with induction therapy for newly diagnosed *FLT3*^mut^ AML, and a more specific, potent FLT3i, gilteritinib as monotherapy for relapsed/refractory (R/R) *FLT3*^mut^ AML have improved outcomes in patients with *FLT3*^mut^ AML. Nevertheless, the short duration of remission with single-agent FLT3i’s in R/R *FLT3*^mut^ AML in the absence of ASCT, limited options in patients refractory to gilteritinib therapy, and diverse primary and secondary mechanisms of resistance to different FLT3i’s remain ongoing challenges that compel the development and rapid implementation of multi-agent combinatorial or sequential therapies for *FLT3*^mut^ AML.

## Overview

Fms-like tyrosine kinase 3 (*FLT3*) is a recurrent genetic abnormality in AML (~30%)^1–3^. *FLT3* activating mutations (*FLT3*^mut^) may involve either the juxta membrane domain [internal tandem duplication mutations (*FLT3*-ITD)]^4^ or the tyrosine kinase domain (*FLT3*-TKD)^5,6^. In patients with newly diagnosed AML, *FLT3-*ITD^mut^ is a poor prognostic factor in terms of relapse-free (RFS) and overall survival (OS)^7–10^. *FLT3*-TKD activating mutations also constitutively activate FLT3^11^; however, they have not been associated with a consistent prognostic impact^12^.

## Prognostic impact of FLT3 mutations

Not all *FLT3-*ITD^mut^ are equal; the prognostic impact is influenced by the allele ratio (AR), insertion site, ITD length, co-mutations (*NPM1*), and karyotype. AR is defined as the ratio of ITD-mutated alleles to wild-type allele (*FLT3*‐ITD/*FLT3* wild-type)^13^. Variant allele frequency (VAF) is the ratio of ITD-mutated alleles to ITD-mutated + wild-type alleles (*FLT3*‐ITD/*FLT3*‐ITD + *FLT3* wild-type)^14^. Schlenk et al. evaluated the impact of AR in 323 patients with newly diagnosed *FLT3*-ITD^mut^ AML. After post-remission therapy with either consolidation (high-dose cytarabine-based) or allogeneic stem cell transplant (ASCT), AR ≥0.51 and *FLT3*-ITD insertion site in TKD1 were associated with an unfavorable RFS (*P* = 0.0008) and OS (P = 0.004)^15^. In fact, every quartile increase in *FLT3*-ITD AR (from 0.01 to 0.20, 0.20 to 0.53, 0.53 to 0.80, 0.80 to 1.19) was associated with worsening complete remission (CR) rates, RFS, and OS, highlighting the prognostic value of AR. It is important to note that none of these patients received a FLT3 inhibitor (FLT3i) during induction, consolidation, or post-ASCT.

The UKMRC group evaluated the presence of *NPM1* co-mutations in young adult patients with AML. Favorable relapse risk and OS were seen in *NPM1*^mut^ with *FLT3* wild-type; intermediate prognosis in *FLT3*-ITD^mut^ with concurrent *NPM1*^mut^, and adverse prognosis in *FLT3*-ITD^mut^ with *NPM1* wild-type patients^16^. The Spanish group evaluated intermediate-risk AML patients treated with intensive chemotherapy. In patients with concurrent *NPM1*^mut^, the OS and relapse risk were comparable between *FLT3* wild-type and *FLT3*-ITD^mut^ AR <0.5, but worse when AR ≥0.5. Among those with *NPM1* wild-type, all *FLT3*-ITD^mut^ patients had an increased risk of relapse and inferior OS, regardless of the AR^17^. The current European Leukemia Net (ELN) guidelines categorize *FLT3* -ITD^mut^ AML as favorable (*NPM1*^mut^ with *FLT3* wild-type Or *NPM1*^mut^ with *FLT3*-ITD AR<0.5), intermediate (*NPM1*^mut^ with *FLT3*-ITD AR>0.5 Or *NPM1*^WT^ with *FLT3*-ITD AR<0.5), or adverse (*NPM1*^WT^ with *FLT3*-ITD AR>0.5)^18^. However, a subsequent UKMRC study of 1600 patients with cytogenetic intermediate-risk AML showed that relapse risk did not differ based on the *FLT3*-ITD^mut^ AR, and that the cumulative incidence of relapse in patients with *NPM1*^mut^ was increased with a concurrent *FLT3*-ITD^mut^ irrespective of the AR^19^. Oran et al. recently showed that ASCT in CR1 improved RFS and OS independent of the *FLT3*-ITD^mut^ AR or *NPM1*^mut^ status in patients with *FLT3*-ITD^mut^ AML^20^. Collectively, *NPM1*^mut^ even with *FLT3*-ITD^mut^ AR <0.5 are likely higher risk than truly “favorable risk” AML and we continue to consider them for ASCT in CR1.

Minetto and colleagues retrospectively evaluated the efficacy of fludarabine, high-dose cytarabine, and idarubicin (FAI) in 149 newly diagnosed *FLT3*-ITD^mut^ and/or *NPM1*^mut^ AML (only *FLT3*-ITD^mut^ = 29; *FLT3*-ITD^mut^ *NPM1*^mut^ = 59, only *NPM1*^mut^ = 61). In patients ≤55 years, this regimen appeared to overcome the negative impact of *FLT3*-ITD^mut^ in *NPM1* co-mutated patients, regardless of the *FLT3* AR, with comparable 3-year OS rates of 64% and 68% in *FLT3*-ITD^mut^ *NPM1*^mut^ and *FLT3*-ITD^WT^ *NPM1*^mut^ patients, respectively (*P* > 0.05). Moreover, ASCT in CR1 only benefitted patients with isolated *FLT3-*ITD^mut^ (without *NPM1*^mut^) irrespective of AR (*P* < 0.05)^21^.

Taken together, utilizing baseline *FLT3*-ITD^mut^ AR to guide the post-remission therapy remains controversial. We currently recommend the incorporation of FLT3i’s and ASCT in CR1 in all ASCT eligible patients with a *FLT3-*ITD^mut^ AML, irrespective of the AR and/or *NPM1* co-mutation status. However, emerging data does suggest that patients with *FLT3*-ITD^mut^ AR<0.5 and *NPM1* co-mutation without concurrent high-risk mutations such as *DNMT3A, TP53, TET2*, or high-risk cytogenetics may be a more favorable subset, who may be considered for induction, consolidation followed by maintenance therapy without ASCT on a case by case basis if they achieve early MRD negativity using a highly sensitive MRD assay.

## First-generation FLT3 inhibitors

Type I FLT3i’s are active against both the FLT3-ITD or TKD, type II inhibitors are only active against FLT3-ITD, not TKD. The first-generation FLT3i’s lack specificity for FLT3 and inhibit multiple downstream RTKs that may result in more off-target toxicities. Second-generation FLT3i’s potently and specifically target FLT3 with fewer off-target effects.

Midostaurin is a type I FLT3i active against PDGFR, KIT, SRC, and other RTKs^22,23^. In the randomized phase III RATIFY trial of midostaurin combined with cytarabine and daunorubicin (3 + 7) induction and consolidation, midostaurin improved OS compared to placebo in patients <60 years of age with newly diagnosed *FLT3 (*ITD and/or TKD*)* AML^24^, regardless of AR (≤0.7 or ≥0.7) or the type of mutation (ITD or TKD). Patients treated with midostaurin had higher rates of anemia and skin rash compared to placebo and these were generally manageable with supportive care without necessitating dose reductions or interruptions in the majority of cases. Pulmonary infiltration and acute pneumonitis-like picture are rare (<1%) but noted side effects of midostaurin that treating physicians should be aware of. Midostaurin has been approved and widely used in combination with induction and consolidation therapy in patients with newly diagnosed *FLT3*^mut^ AML^25^.

Sorafenib is a first generation, type II multi-kinase FLT3i^26^ that demonstrated safety and efficacy (14/15 CR) in combination with the standard anthracycline/cytarabine induction therapy in newly diagnosed *FLT3*^mut^ AML^27^. SORAML, a randomized placebo-controlled trial evaluated the efficacy and tolerability of 3 + 7 induction-consolidation with or without sorafenib in patients ≤60 years with newly diagnosed AML, irrespective of a *FLT3*^mut^ (only 34% had *FLT3*^mut^). The addition of sorafenib significantly improved the event-free survival (EFS; 21 months vs 9 months; *P* = 0.013) and RFS (56% vs 38%), but not OS^28^, although a recent update suggested an emerging trend toward improved OS^29^. The sorafenib treatment arm had increased rates of adverse events, particularly diarrhea, bleeding, cardiac events, hand-foot-skin reaction, and rash but with no significant increase in the 30- or 60-day mortality between the two treatment arms. Intriguingly, this was the first large study to show that the FLT3i may also benefit *FLT3* wild-type patients, perhaps through multi-kinase blockade or prevention of emergent *FLT3* clones at relapse^28^. Recently, a double-blind placebo-controlled study reported a trend toward improved OS but not EFS with sorafenib combined with intensive chemotherapy in the frontline setting, especially among those with high *FLT3*-ITD^mut^ AR >0.7^30^. Sorafenib with azacitidine combination reported an overall response rate (ORR) of 78% (*n* = 27) in the frontline patients not eligible for intensive induction^31^ and an ORR of 46% with an acceptable safety profile in R/R *FLT3*-ITD^mut^ ^32^ which led to the inclusion of sorafenib with azacitidine combination as a 2B guideline in National Comprehensive Cancer Network (NCCN) for R/R *FLT3-*ITD^mut^ AML^33^.

## Second-generation FLT3 inhibitors

Quizartinib, a second-generation, type I FLT3i is active against FLT3, KIT, CSF1R, PDGFR, and RET kinase^34^. Unlike midostaurin, quizartinib monotherapy, even at lower doses demonstrated significant marrow remissions in R/R *FLT3*^mut^ AML^35–37^. In a single-arm phase II trial of quizartinib (90 or 135 mg), the CRc rates were between 46 and 56% in ~250 R/R *FLT3*-ITD^mut^ patients treated across two cohorts. QTc prolongation >500 ms emerged as a significant adverse event^36^. A subsequent randomized phase IIb trial evaluated lower doses, 30 or 60 mg of quizartinib daily, in patients with R/R *FLT3-*ITD^mut^ AML. The CRc rates (47%) were similar between both doses, and the frequency of QTcF >500 ms was significantly reduced (3–5%) with these lower doses of quizartinib^35^.

QuANTUM-R, a phase 3 randomized controlled trial, evaluated quizartinib monotherapy vs investigator choice salvage chemotherapy in R/R *FLT3-ITD*^mut^ AML. Quizartinib demonstrated an OS of 6.2 months compared with 4.7 months with salvage chemotherapy (hazard ratio 0.76 and *P* = 0.02). The CRc rates with quizartinib were similar to prior studies (48.2%), and 32% patients on the quizartinib arm underwent ASCT compared with 11% with salvage chemotherapy. Given the magnitude of OS benefit and concerns over therapeutic equipoise and potential cardiac safety signals, quizartinib was not approved in the US and Europe, but approved in Japan as a monotherapy in R/R *FLT3-ITD*^mut^ AML. In general, quizartinib is well tolerated with minimal skin, gastrointestinal, or pulmonary side effects. However, in addition to QTcF prolongation, quizartinib is also more myelosuppressive than many other FLT3 inhibitors likely due to the inhibition of KIT.

Gilteritinib, a second-generation type I FLT3i demonstrated tolerability with CRc rates of 45–55% in patients with R/R *FLT3 (ITD or TKD)*^mut^ AML^38,39^. The randomized phase III ADMIRAL trial evaluated gilteritinib vs investigator choice salvage chemotherapy in patients with R/R *FLT3*^mut^ AML. Gilteritinib decreased the risk of death by 36% compared with salvage chemotherapy, with a median OS of 9.3 months vs 5.6 months (*P* < 0.001), and a superior CR+CRh rate (34% vs 15.3%). Gilteritinib was generally well tolerated but was associated with increased incidence of gastrointestinal side effects, most frequently diarrhea although nausea has been occasionally observed. Increase in bilirubin and transaminase can be seen with giltertiinib but are usually self-resolving and transient. Posterior reversible encephalopathy and pancreatitis are rare (<1–2%) but important side effects to be aware of. These results led to the approval of gilteritinib monotherapy in the US and Europe in patients with R/R *FLT3*^mut^ AML^40^.

## Post-ASCT maintenance with FLT3 inhibitors

In patients with *FLT3*^mut^ AML who relapsed after first ASCT, sorafenib was found to be tolerable with long-lasting remissions in 7 of 29 patients treated, suggesting a potential synergy with post-ASCT alloimmune effects^41^. SORMAIN, a placebo-controlled randomized phase II trial evaluated post-transplant sorafenib maintenance in patients with *FLT3-ITD*^mut^ AML with RFS post-ASCT as the primary endpoint. At a median follow-up of 42 months, sorafenib demonstrated a 2-year estimated RFS of 85% and OS of 90.5% compared with 53.3% (*P* = 0.002), and 66.2% with placebo, respectively (*P* = 0.007). Although the toxicity-related discontinuation rate was low (22%), sorafenib-treated patients did experience higher rates of graft-versus-host disease (GVHD) and skin toxicity^42^. In another randomized phase III study comparing post-ASCT sorafenib maintenance (*n* = 100) to non-maintenance (*n* = 102), sorafenib demonstrated an improved 1-year OS (82.1% vs 68%, *P* = 0.012) and a decreased 1-year cumulative incidence of relapse (7% vs 24.5%, *P* = 0.001) in *FLT3-ITD*^mut^ AML patients undergoing ASCT in CR1^43^. We currently recommend post-transplant maintenance with a FLT3i for at least 2 years (potentially indefinitely as there is limited data on the incidence of possible late relapses) in all *FLT3*^mut^ AML. The MORPHO phase III placebo-controlled trial evaluating post-transplant maintenance with gilteritinib in *FLT3*^mut^ AML recently completed accrual and results are eagerly awaited (NCT02997202).

## Moving forward to maximize benefit: FLT3 inhibitors combination therapy

### Frontline FLT3i’s with anthracycline/cytarabine induction or hypomethylating agents (HMAs)

A phase I study evaluating gilteritinib with 7 + 3 induction and high-dose cytarabine consolidation chemotherapy, followed by single-agent maintenance therapy, in patients with newly diagnosed AML showed that gilteritinib 120 mg daily was well tolerated. Among 38 patients with *FLT3*^mut^ AML who received gilteritinib 120 mg daily, the CRc rate was 81.6% (*n* = 31) including 39.5% CR and median OS was not reached at a median follow-up of 35.8 months. Two randomized trials are evaluating the addition of gilteritinib vs midostaurin to induction and consolidation therapy in patients with newly diagnosed *FLT3*^mut^ AML^44^ (NCT04027309, NCT03836209).

The LACEWING phase III randomized trial evaluated gilteritinib with azacitidine vs azacitidine monotherapy (NCT02752035) in patients with newly diagnosed *FLT3*^mut^ AML not eligible for intensive induction chemotherapy. The CRc rate was 67% (*n* = 10/15) in the combination arm in the safety cohort prior to commencement of randomization^45^. However, in a recently released planned interim analysis, the study did not meet its primary endpoint of overall survival and may be terminated for futility^46^.

Strati et al. evaluated midostaurin with azacitidine in patients with both newly diagnosed and R/R AML regardless of *FLT3* mutational status. Among the *FLT3*^mut^ patients, response rates were numerically higher (33%) and remission duration was longer (31 versus 16 weeks, *P* = 0.09) in those who were naive to treatment with FLT3 inhibitors compared with those who had been exposed to prior FLT3 inhibitors. Although activity was seen, the response rates were overall modest with this combination and the combination of HMA with midostaurin is not one that we routinely use or recommend for frontline FLT3-mutated AML^47^.

Swaminathan et al. evaluated quizartinib (60 mg daily) combined with either azacitidine or low-dose cytarabine in patients with newly diagnosed or R/R *FLT3*^mut^ AML not eligible for intensive chemotherapy. Among patients treated with azacitidine and quizartinib in the frontline setting, the CRc rate was 78% (*n* = 7/9) with a median OS of 21.1 months. In the R/R setting, the CRc rate was 64% (*n* = 18/28) with a median OS of 12.0 months, with responses observed even in prior FLT3i exposed patients^48^. A randomized, placebo-controlled phase III study of 3 + 7 with quizartinib (QuANTUM-First; NCT02668653) in patients with newly diagnosed *FLT3-ITD*^mut^ AML eligible for induction therapy recently completed accrual. Quizartinib is also being evaluated in combination with CPX-351 (NCT04209725) and with CLIA (NCT04047641) in treatment naive and R/R *FLT3*^mut^ AML.

### Combinations with venetoclax with or without HMA

Based on the strong preclinical synergy and synthetic lethality with venetoclax and FLT3i combination^49–51^, and the fact that BCL2 upregulation may confer resistance to FLT3 inhibition^52^, evaluation of several doublet and triplet combinations of venetoclax and FLT3i are ongoing. Gilteritinib with venetoclax (NCT03625505) was evaluated in 41 patients with heavily pretreated R/R *FLT3*^mut^ AML (median salvage 2, 65% previously exposed to FLT3i)^40,53^. Using the same response criteria, the CRc rate was 85.4% (*n* = 35/41) which compared favorably to 52% with gilteritinib alone in the ADMIRAL study. However, the true CR/CRi rate was only 34%. Molecular clearance of *FLT3* was noted in 50% of all evaluable patients. Encouragingly, the response rate was maintained among patients previously exposed to other FLT3 TKIs. The combination continues to enroll.

Maiti et al. recently presented the first triplet combination of venetoclax, FLT3i (mainly gilteritinib or sorafenib), and decitabine from the *FLT3*^mut^ subset of the prospective decitabine 10 days with venetoclax study (NCT03404193)^54^. Among 16 patients with newly diagnosed *FLT3*^mut^ AML not eligible for intensive induction, the CRc rate was 88% with *FLT3*-PCR negativity in 100% of responders and a projected 2-year OS of >80%. Among 14 R/R *FLT3*^mut^ AML patients, the CRc rate was 64% with *FLT3*-PCR negativity in 88% of responders. In the treatment-naive setting, the median time to neutrophil and platelet recovery among responders was 45 and 30 days, respectively, suggesting cumulative myelosuppression is to be expected and further optimization of triplets schedules is ongoing^55^.

Yilmaz et al. prospectively evaluated decitabine and quizartinib (doublet) with or without venetoclax (triplet) in patients with newly diagnosed and R/R *FLT3-*ITD^mut^ AML. While the seven patients treated with the doublet had a CRc rate of 57% (*n* = 4/7) and a median OS of 5.7 months, the fifteen R/R *FLT3*^mut^ AML patients treated with the triplet had a CRc rate of 81% (*n* = 11) with a projected 1-year OS of 60%. In the frontline setting (*n* = 4), the CRc rate with the triplet was 100% with *FLT3*-PCR negativity in all four patients^56^.

These data highlight the potent anti-leukemic activity of the triplet approach in *FLT3*^mut^ AML. We believe that triplets may be the optimal way to use FLT3i to improve long-term survival and “cure rates” in older patients, able to tolerate this approach. Further evaluation and optimization of triplets is a major area of clinical research focus in *FLT3*^mut^ AML.

### Treatment algorithm of *FLT3*-mutated AML

**Fig. 1:**
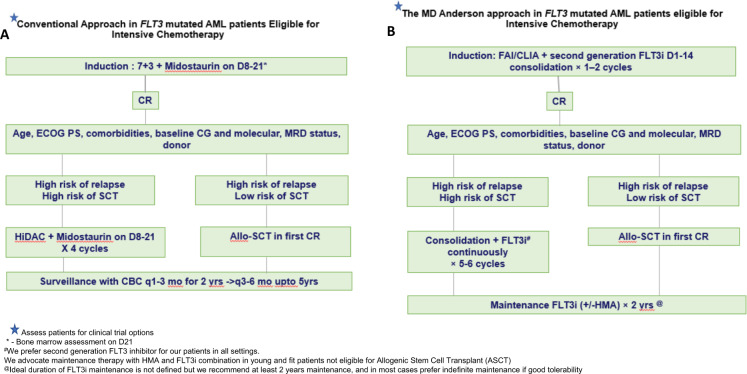
Treatment algorithm of *FLT3*-ITD-mutated AML in patients eligible for intensive chemotherapy. **A** Conventional approach. **B** MD Anderson Cancer Center Approach. 7+3—7 days of cytarabine and 3 days of daunorubicin. F fludarabine, I idarubicin, CL cladribine, A cytarabine 1.5–2 g/m^2^, HMA hypomethylating agent, CR complete remission, ECOG PS Eastern Cooperative Oncology Group Performance Status, CG cytogenetics, MRD measurable residual disease, SCT stem cell transplant, HiDAC high-dose cytarabine, CBC complete blood count.

Clinical trial enrollment (if available) is always the first option, in both frontline and R/R *FLT3*^mut^ AML. The choice of treatment backbone depends on the patient’s ability to successfully tolerate intensive chemotherapy. Accumulating evidence have shown improved outcomes in *FLT3*-ITD^mut^ patients receiving induction with higher dose anthracyclines^57^, cladribine^58^, or fludarabine added to induction backbone^21^, and incorporating FLT3i with induction (either first or second generation) in *FLT3*^mut^ AML^24,44,59,60^ (Fig. 1A). Our treatment approach for *FLT3*^mut^ AML in MD Anderson Cancer Center is as follows: in newly diagnosed patients who are eligible to receive intensive chemotherapy (Fig. 1B) we add a second generation FLT3i to the intensive induction backbone of cladribine or fludarabine with cytarabine and idarubicin (CLIA or FIA, respectively) as published previously by our group^61,62^. Addition of venetoclax to this backbone may be associated with prolonged and potentially prohibitive myelosuppression; we have not routinely added and do not at this time recommend adding venetoclax to the backbone of CLIA/FIA with FLT3i^63^. We prefer a second-generation FLT3i (ideally gilteritinib) in the newly diagnosed setting, and administer the FLT3i D1-D14 during induction, and continuously starting Cycle 2 Day 1 through consolidation.

Upon achieving CR, the decision for ASCT is based on the risk-benefit assessment for ASCT. All eligible intermediate or high-risk patients (defined as patients with *FLT3*-ITD^mut^ AR>0.50 irrespective of *NPM1*^mut^ status, or *FLT3*-ITD^mut^ AR<0.50 without *NPM1*^mut^) are equivocally recommended to proceed to ASCT in CR1 followed by post-ASCT FLT3i maintenance for at least 2 years (although we often continue indefinite FLT3i maintenance until long-term maintenance data becomes available). For post-ASCT maintenance, our agent of choice has been gilteritinib 80–120 mg day either as a single agent or combined with low-dose azacitidine. The role of ASCT in patients with *FLT3*-ITD^mut^ AR<0.50 with concomitant *NPM1*^mut^ in the absence of concomitant high-risk features such as *DNMT3A, TP53*, or *RUNX1* co-mutations, adverse cytogenetics, therapy-related or secondary AML, who achieve MRD negativity by high-sensitivity PCR (ideally for *NPM1*^mut^), or patients with *FLT3*-TKD^mut^ is an area of ongoing debate. We evaluate these patients on a case by case basis and may consider maintenance with 4–5 consolidation cycles of CLIA or FAI with FLT3i followed by FLT3i +/− HMA maintenance for two years vs ASCT based on donor availability, age, performance status, MRD negativity, and patient preference.

**Fig. 2:**
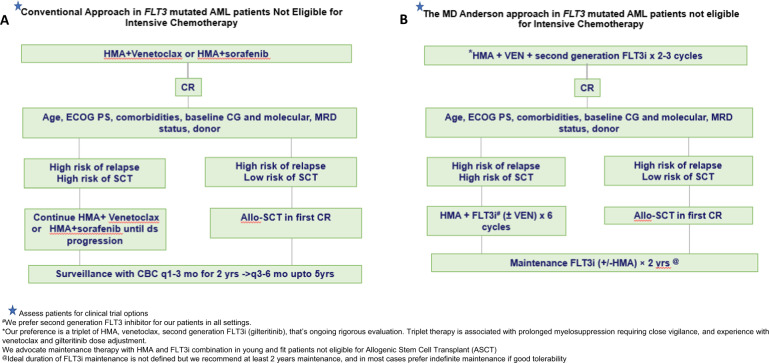
Treatment algorithm of *FLT3*-mutated AML in patients not eligible for intensive chemotherapy. **A** Conventional approach. **B** MD Anderson Cancer Center Approach. FLT3i FLT3 inhibitor, HMA hypomethylating agent, VEN venetoclax, CR complete remission, ECOG PS Eastern Cooperative Oncology Group Perfromance Status, CG cytogenetics, MRD measurable residual disease, SCT stem cell transplant, CBC complete blood count.

In patients with *FLT3*^mut^ AML unsuitable for intensive chemotherapy, azacitidine with venetoclax demonstrated encouraging CR/CRi rates (55–70%) and a median OS of 13.3 months^64^ which prompted the inclusion of this combination approach as part of NCCN AML guidelines (Fig. 2A). However, the median OS was 19.2 months in *FLT3*-TKD^mut^ AML (19.2 months), but only 11.5 months in *FLT3*-ITD^mut^ patients^65^. This is in line with the preclinical data^49^ and molecular profiling of pre- and post-treatment samples^66^ identifying *FLT3*-ITD^mut^ as a putative mechanism of resistance to venetoclax based therapies^67^, suggesting that *FLT3*-ITD^mut^ patients may need a FLT3i incorporated into the HMA with venetoclax therapy either in a triplet or sequential approach to improve OS. Therefore, in patients not eligible for intensive chemotherapy at MDACC, we prefer a combination of HMA with venetoclax and FLT3i (gilteritinib) over an HMA with venetoclax doublet (Fig. 2B). Administration of the triplet is associated with prolonged cytopenias, requiring close monitoring and experience with venetoclax based combinations.

**Fig. 3:**
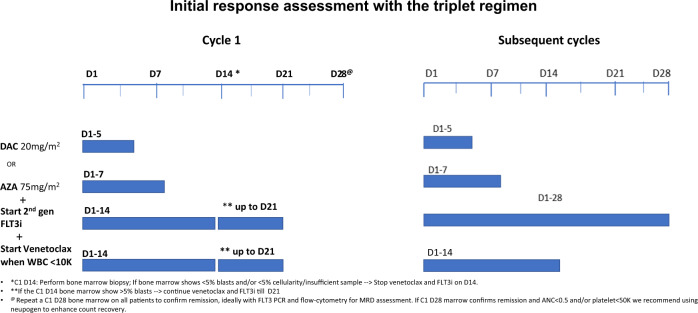
Initial response assessment with the triplet regimen. *C1 D14: Perform bone marrow biopsy; if bone marrow shows <5% blasts and/or <5% cellularity/insufficient sample → Stop venetoclax and FLT3i on D14. **If the C1 D14 bone marrow show >5% blasts → continue venetoclax, FLT3i till D21. ^@^Repeat a C1 D28 bone marrow on all patients to confirm remission^.^ If C1 D28 marrow confirms remission and ANC<0.5 and/or platelet < 50K consider interrupting FLT3i and using neupogen to enhance count recovery.

We administer a second-generation FLT3i (ideally gilteritinib) continuously with HMA from cycle 1 Day 1. We introduce venetoclax with a ramp-up when the WBC is <10,000/µL to decrease the risk of tumor lysis syndrome. To mitigate prolonged myelosuppression with the triplet and avoid over-treatment, we perform an early bone marrow assessment on Cycle 1 Day 14 (Fig. 3). We stop the venetoclax and the FLT3i after Day 14 in patients who achieve marrow remission (<5% blasts) and/or marrow aplasia/hypoplasia/insufficiency (<5% cellularity). We continue the venetoclax and FLT3i until Day 21 if the Day 14 bone marrow shows >5% blasts with >/=5% cellularity. In patients with ongoing cytopenias (ANC</=0.5 and/or platelets </=50K) on Day 28, we repeat a bone marrow on Day 28 to confirm marrow remission and once confirmed recommend administering growth factors starting Day 28 to boost recovery. In subsequent cycles: FLT3i is continued for the entire duration of the cycle and the venetoclax duration is reduced to 14 days or lower to mitigate cumulative prolonged cytopenias. Although the triplet approaches are still in development, emerging data with the triplets as discussed previously, suggest rapid and high potency, deep molecular remissions, and encouraging survival. An alternate option would be to consider sequencing with alternate cycles of HMA with venetoclax and HMA with FLT3i. Such sequential approaches need to be formally evaluated in the context of prospective clinical trials.

**Fig. 4:**
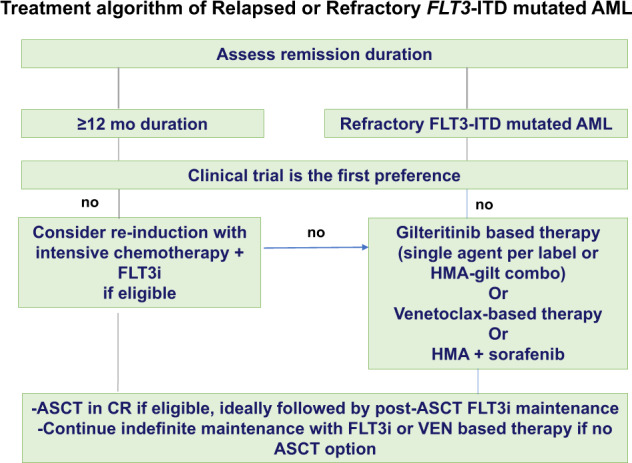
Treatment algorithm of relapsed or refractory *FLT3*^mut^ AML. SCT stem cell transplant.

In patients with relapsed or refractory *FLT3*^mut^ AML (Fig. 4), diligent effort must be made to refer the patient to large academic centers with clinical trial options as the outcomes remain dismal with a median OS < 10 months with almost any approach. In the absence of clinical trial options: among patients eligible for intensive chemotherapy who had a prior remission >10–12 months, we would prefer a regimen incorporating intensive therapy (FLAG-Ida, CLAG-M, CLIA, MEC) in combination with a FLT3 inhibitor with an intent to achieve a rapid and hopefully deep remission and transition patients to ASCT followed by post-ASCT maintenance. In older patients not eligible for intensive therapy, patients with primary refractory disease or early relapse with a persistent *FLT3* mutation we would suggest gilteritinib based therapy. Although the label indication for gilteritinib is as a single agent we have never used it as a single agent but always in combination with either HMA alone, venetoclax alone or as a triplet with HMA and venetoclax. These combinations appear to improve the efficacy over single agent gilteritinib and could be considered if there is expertise in using such an approach, For patients relapsing while on gilteritinib or soon after gilteritinib based therapy a combination of azacitidine with sorafenib or azacitinde with venetoclax or gemtuzumab based approaches may be considered as salvage options (with clinical trials being clearly the best option if available).

### Mechanisms of resistance to FLT3 inhibitors

Despite the encouraging development of FLT3i, resistance to FLT3i is not uncommon and it can be either primary or secondary. The primary resistance mechanisms include specific *FLT3*-TKD mutations (either single TKD mutations or compound mutations within the *FLT3*-ITD allele), mutations in genes other than *FLT3*, activation of alternative signaling pathways in leukemic cells or the bone marrow microenvironment that confer resistance to FLT3i^68^.

Secondary resistance to FLT3i could be either on-target (changes in the FLT3) or off-target (constitutive activation of non-FLT3-dependent oncogenic pathways). The on-target mechanism of resistance includes emergence of secondary TKD mutations in patients treated with type II inhibitors like quizartinib or sorafenib^69,70^. Type I FLT3i’s like gilteritinib are less prone to develop secondary mutations in the TKD, although the gatekeeper F691M can confer resistance to gilteritinib^71^. Off-target resistance includes clonal evolution during FLT3i therapy even when *FLT3*-ITD^mut^ clone is lost^70^. In a study that identified molecular mechanisms of resistance to gilteritinib, 32% of patients had emergent mutations in the RAS/MAPK pathway (*K/NRAS*), and 5% had emergent *BCR/ABL1* fusions^71^. More recently, the emergence of *BCR-ABL1*-positive clone was shown as a resistance mechanism to multiple FLT3i’s^72^. It is important to acknowledge the diverse mechanisms of FLT3i resistance after different FLT3i’s, and it is essential to proactively evaluate for these mechanisms at the time of FLT3i failure to optimize subsequent therapy.

## Future direction

In the QuANTUM-R and ADMIRAL trials, only 4% and 12% of patients had received prior FLT3i therapy with induction, making it difficult to draw conclusions regarding the outcomes of contemporary patients, most of whom will have received a FLT3i (commonly midostaurin) with induction^36,40^. Yilmaz et al. evaluated the outcomes of sequential FLT3i-based therapies in *FLT3*^mut^ AML. In the frontline setting, there was a sequential decrease in CRc rates (77%→31%→25%) and OS (16.7→6.0→1.4 months). A comparable decrease in CRc rates (45%→21%→10%) and OS (7.9→4.0→4.1 months) was observed with sequential FLT3i-based therapies in the R/R AML setting^73^. Perl and colleagues investigated whether prior FLT3i therapy influenced outcomes in patients treated with gilteritinib. Regardless of prior FLT3i therapy, gilteritinib-treated patients had CRc rates >40%, however, the median OS with single-agent gilteritinib was 6.5 vs 9.6 months in prior FLT3i exposed (*n* = 31) vs naive patients (*n* = 216) with *FLT3*^mut^ R/R AML^74^. These data suggests that although responses may still be achieved with gilteritinib in patients refractory to prior first-generation FLT3i-based therapies, optimization with doublet or triplet combinations with second-generation FLT3i is likely needed to significantly improve OS with prior TKI exposure.
